# Supplementary material for: The H3K36me3 methyltransferase SETD2 contributes to PAF1C interactions with RNA Pol II and is required for neuronal differentiation
Source: EMBO J. 2026 Apr 10;45(10):3430–43. doi: 10.1038/s44318-026-00768-2 (PMC13187324; doi:10.1038/s44318-026-00768-2)
Supplement: Supplementary file 1 — Appendix [file 44318_2026_768_MOESM1_ESM.pdf]

Appendix for: The H3K36me3 methyltransferase SETD2 contributes to PAF1C interactions with RNA Pol II and is required for neuronal differentiation

Christina Ambrosi[1,2], Ramon Pfaendler[1,2,a], Kristeli Eleftheriou[3], Stefan Butz[1,2], Davide Recchia[1,2,3], Xue Bao[3], Richard Cardoso da Silva[3], Niklas Kupfer[3], Ilse Lagerwaard[3], Hanneke Vlaming[3], Nina Schmolka[1,b], Vivek Bhardwaj[3], and Tuncay Baubec[1,3,*]

Table of Contents

Appendix Figure S1 - page 2

Appendix Figure S2 - page 3

Appendix Figure S3 - page 4

Appendix Figure S4 - page 6

Appendix Figure S5 - page 8

Appendix Figure S6 - page 10

Appendix Figure S7 - page 11

Appendix Figure S8 - page 13

Appendix Figure S9 - page 15

Appendix Figure S10 - page 16

Appendix Figure S11 - page 18

Appendix Figure S12 - page 20

Appendix Figure S13 - page 22

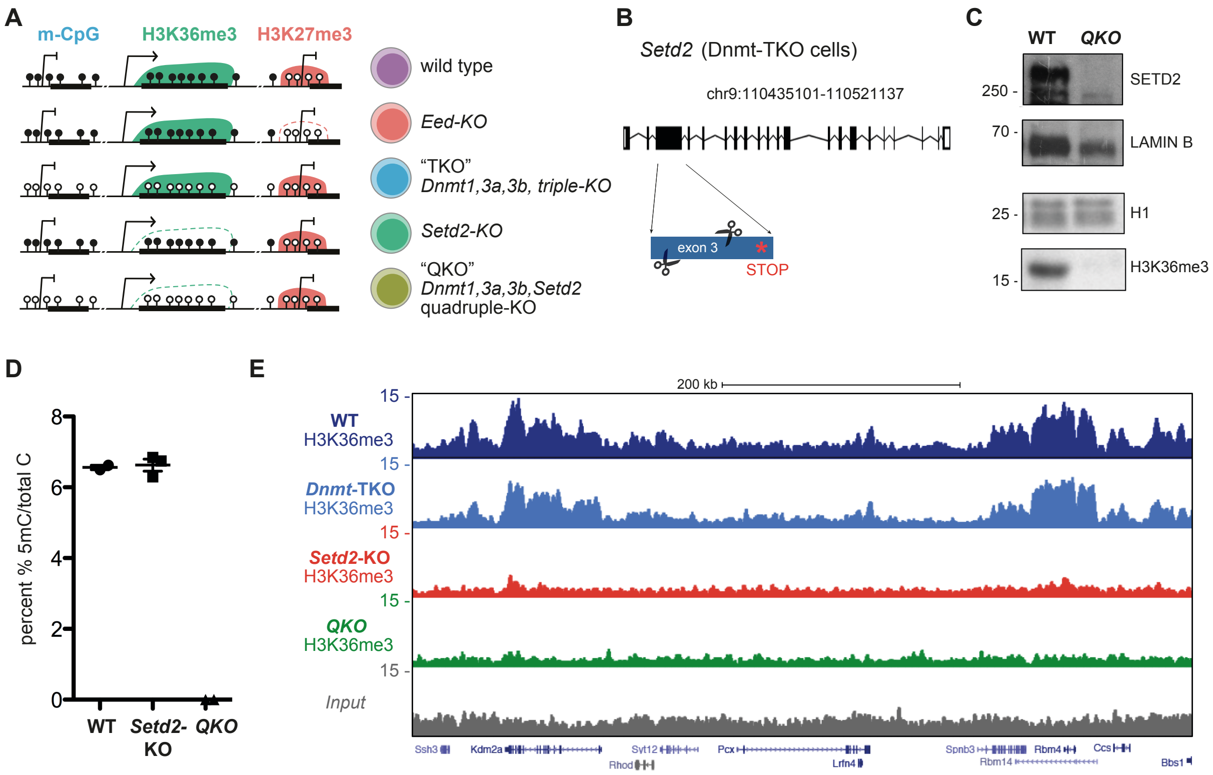

Appendix Figure S1. (A) Schematic overview of mESC lines utilized in this study. Eed-KO lack H3K27me3, Dnmt-TKO lack DNA methylation (5mC), Setd2-KO cells lack H3K36me3, and QKO (Setd2-KO in Dnmt-TKO) cells lack both H3K36me3 and 5mC. **(B)** CRISPR-Cas9 knock-out strategy for Setd2 in Dmnt-TKO background (QKO). Two guide RNAs targeting exon 3, result in an out-of-frame deletion and a downstream premature stop codon. **(C)** Immunoblot analysis for SETD2 and H3K36me3 levels in nuclear (top) and histone (bottom) extracts of wild-type (WT) and QKO mESCs. LAMIN B and H1 serve as loading controls. **(D)** HPLC-MS measurement of % methylcytosine content of cytosine extracted from genomic DNA indicates absence of methylation in the QKO cells. Error bars denote standard deviation from three independent replicate measurements. **(E)** Representative genome browser view of a chromosome 19 locus exemplifying differences in H3K36me3 signals between wild-type, Dnmt TKO, Setd2-KO, and QKO mESCs. Shown are read counts per 100 bp for ChIP-seq and input samples.

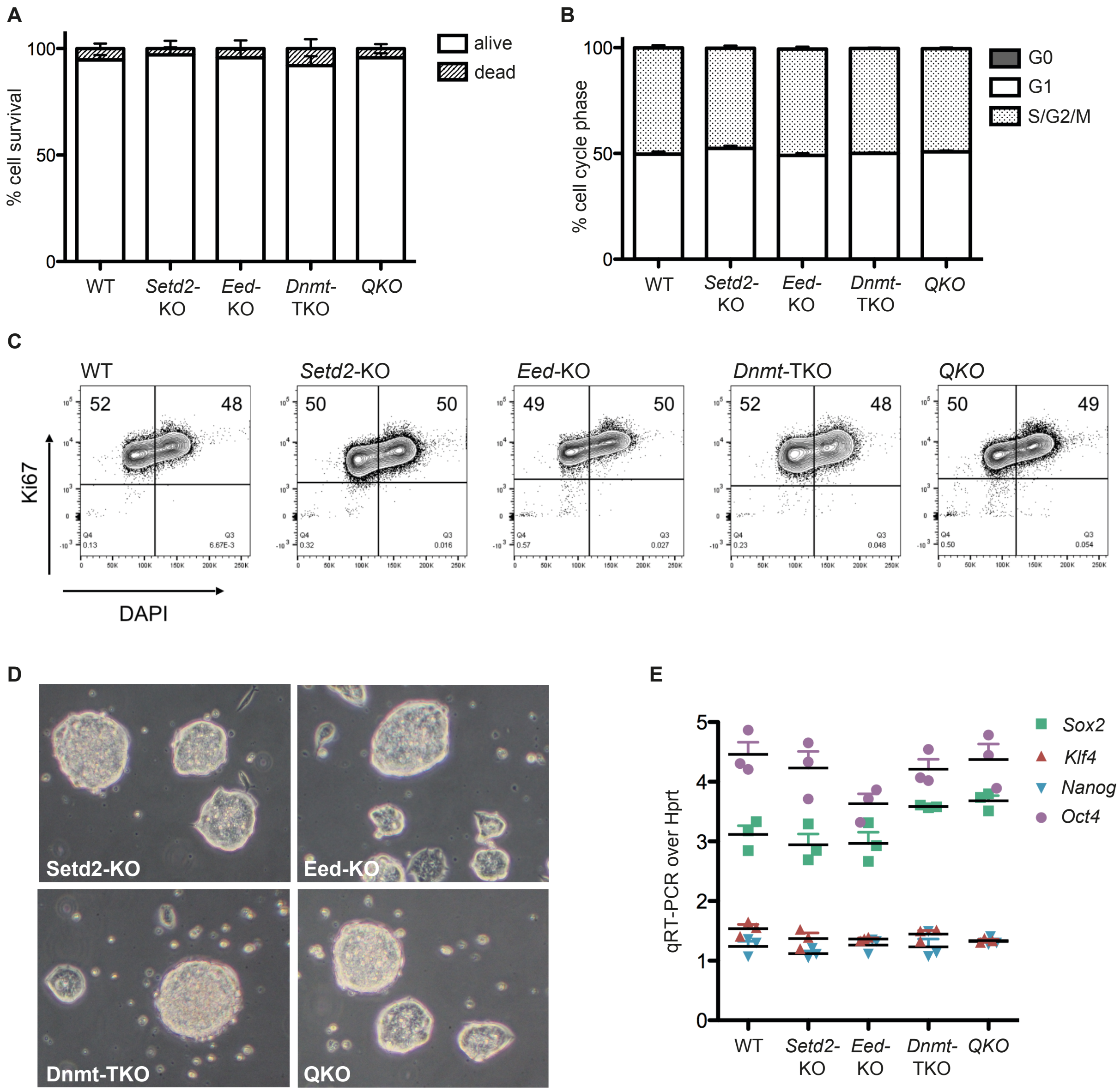

Appendix Figure S2. (A) Wild-type (WT), *Setd2*-KO, *Eed*-KO, *Dnmt*-TKO, and QKO mESCs were cultured in media containing leukemia-inhibitory factor (LIF) over three passages and dead/live cells were counted. **(B)** Ki-67 and DAPI cell cycle analysis by FACS. Shown are percentages of the different phases of WT, *Setd2*-KO, *Eed*-KO, *Dnmt*-TKO and QKO ESCs, based on two independent replicates. **(C)** Exemplary FACS analysis for Ki-67 and DAPI levels in WT, *Setd2*-KO, *Eed*-KO, *Dnmt*-TKO, and QKO mESCs. Highlighted are percentages of cells in Ki67+ (G1 phase) and Ki67+/DAPI+ (S/G2/M phase) clusters. **(D)** Microscopy images of *Setd2*-KO, *Eed*-KO, *Dnmt*-TKO and QKO mESCs in feeder-free culture at 100x magnification. **(E)** RT qPCR detection of pluripotency marker genes *Nanog*, *Pou5F1* (*Oct4*), *Klf4*, and *Sox2 in* WT, *Setd2*-KO, *Eed*-KO, *Dnmt*-TKO, and QKO mESCs. *Hprt* served as internal control.

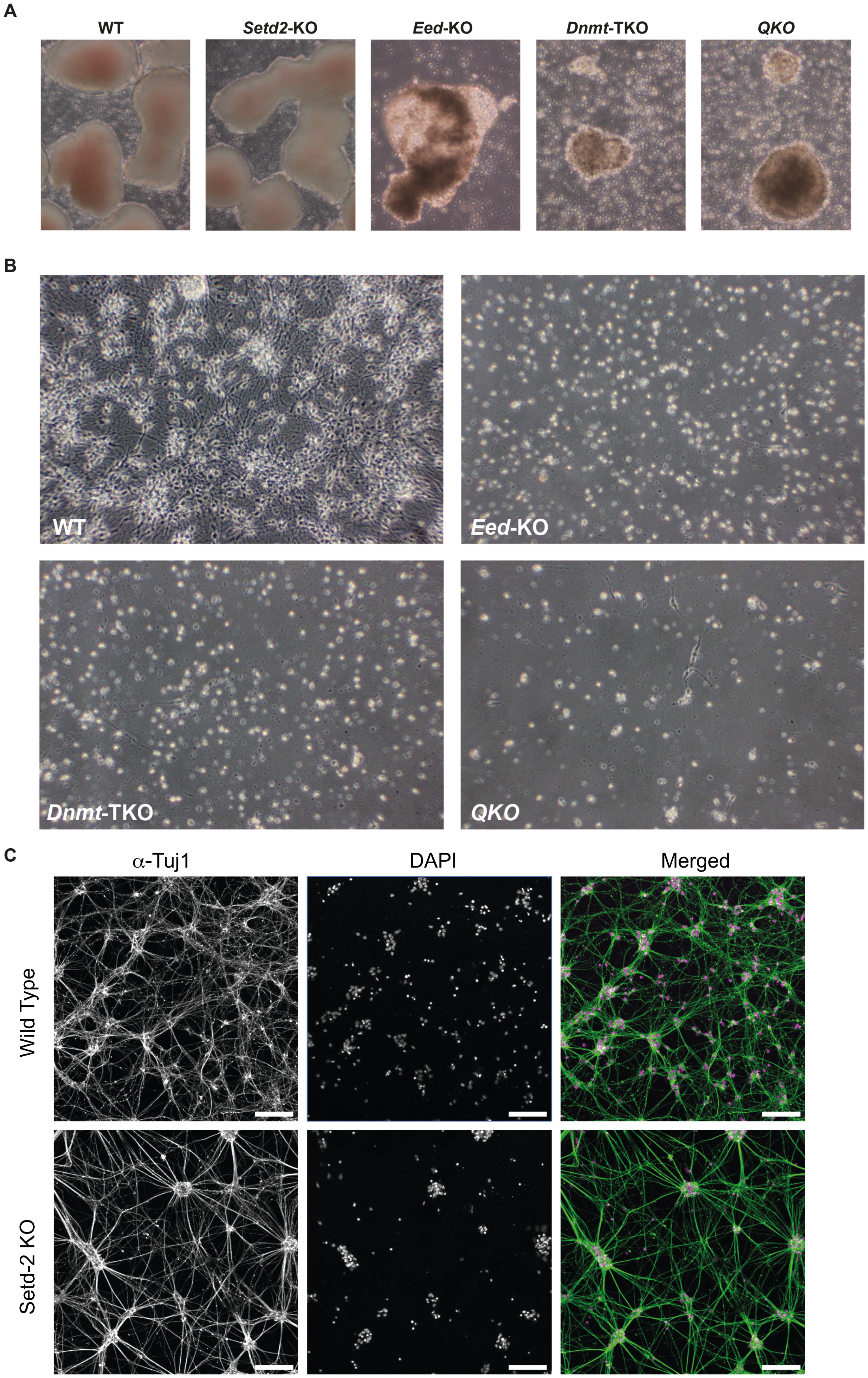

Appendix Figure S3. - Legend on next page

Appendix Figure S3. (A) Exemplary microscopy images of in vitro derived cellular aggregates at day 4 (CAd4) for WT, *Setd2*-KO, *Eed*-KO, *Dnmt*-TKO, and QKO cells at 100x magnification. **(B)** Exemplary microscopy images of in vitro derived, terminal neurons of wild-type, *Eed*-KO, *Dnmt*-TKO and QKO cells at day 14 at 100x magnification. **(C)** Representative immunofluorescence images of TNs generated from wild type and Setd2 KO mESCs after 14 days post neuronal differentiation stained for the neuronal marker Tuj1 (green). Nuclei were visualized with DAPI (magenta). Scale bar = 50 µm. Experiment was repeated twice.

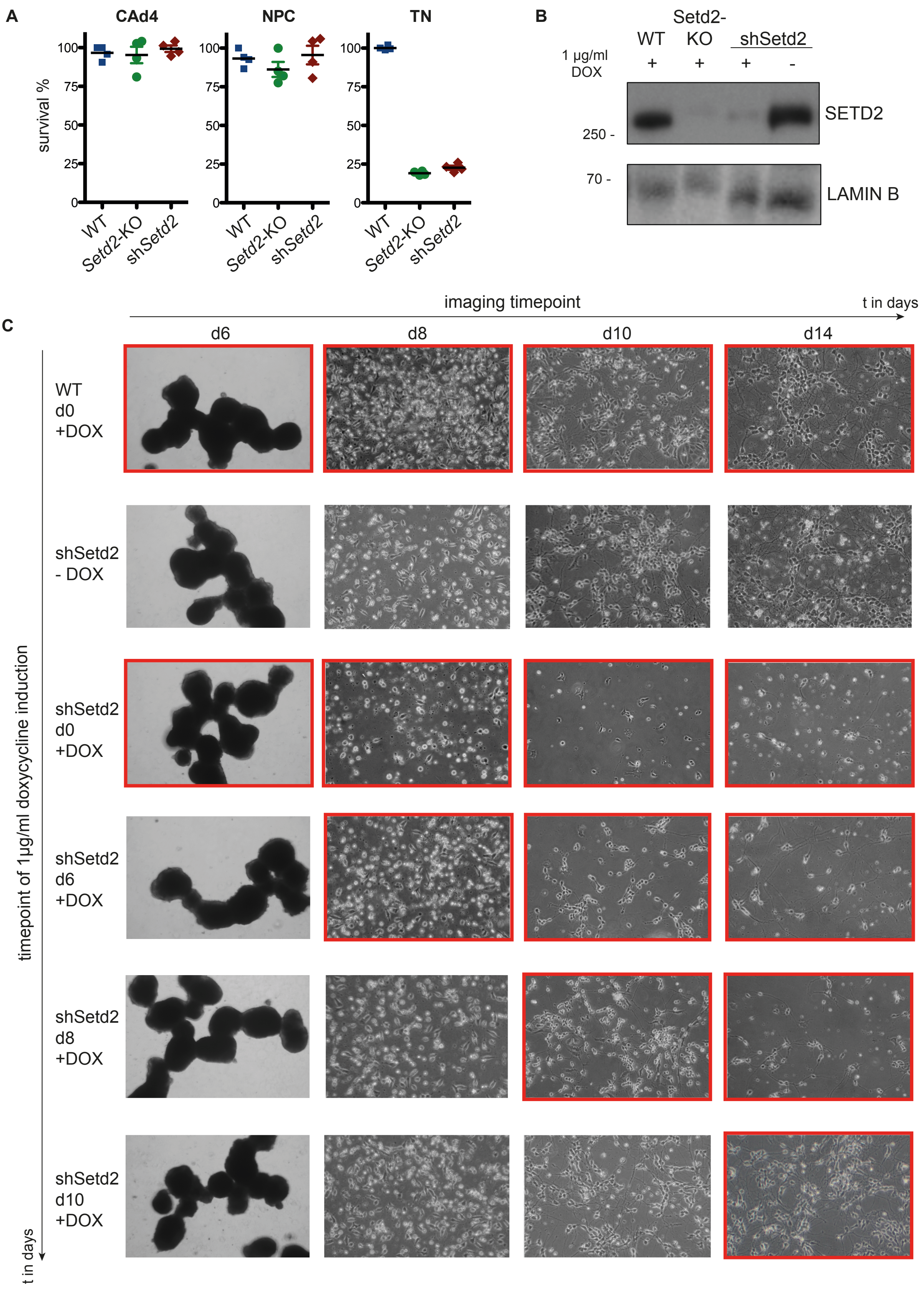

Appendix Figure S4 – legend on next page

Appendix Figure S4. (A) Depicted are percentages of survival in WT, *Setd2*-KO, and cells harboring a constitutively expressed shRNA against *Setd2* (sh*Setd2*) at aggregate stage day 4 (CAd4), neural progenitor cells (NPCs), and terminal neurons (TNs). **(B)** Immunoblot analysis for SETD2 levels in nuclear extracts of WT, *Setd2*-KO, and Tet-inducible shSetd2 knockdown in terminal neurons at day d14 (+/- 1 µg/ml DOX). LAMIN B serves as loading control. **(C)** Time-resolved microscopy images of *in vitro* differentiation using Tet-inducible *Setd2* knockdown cell lines. Shown are images starting from cellular aggregates (CA) day 6 over neural progenitors at day 8 to terminal neurons at day 10 and 14. Treatment with 1 µg/ml doxycycline (DOX) was initiated at day 0, 6, 8, and 9; 100x magnification. Red boxes indicate images taken from neural cells in presence of DOX.

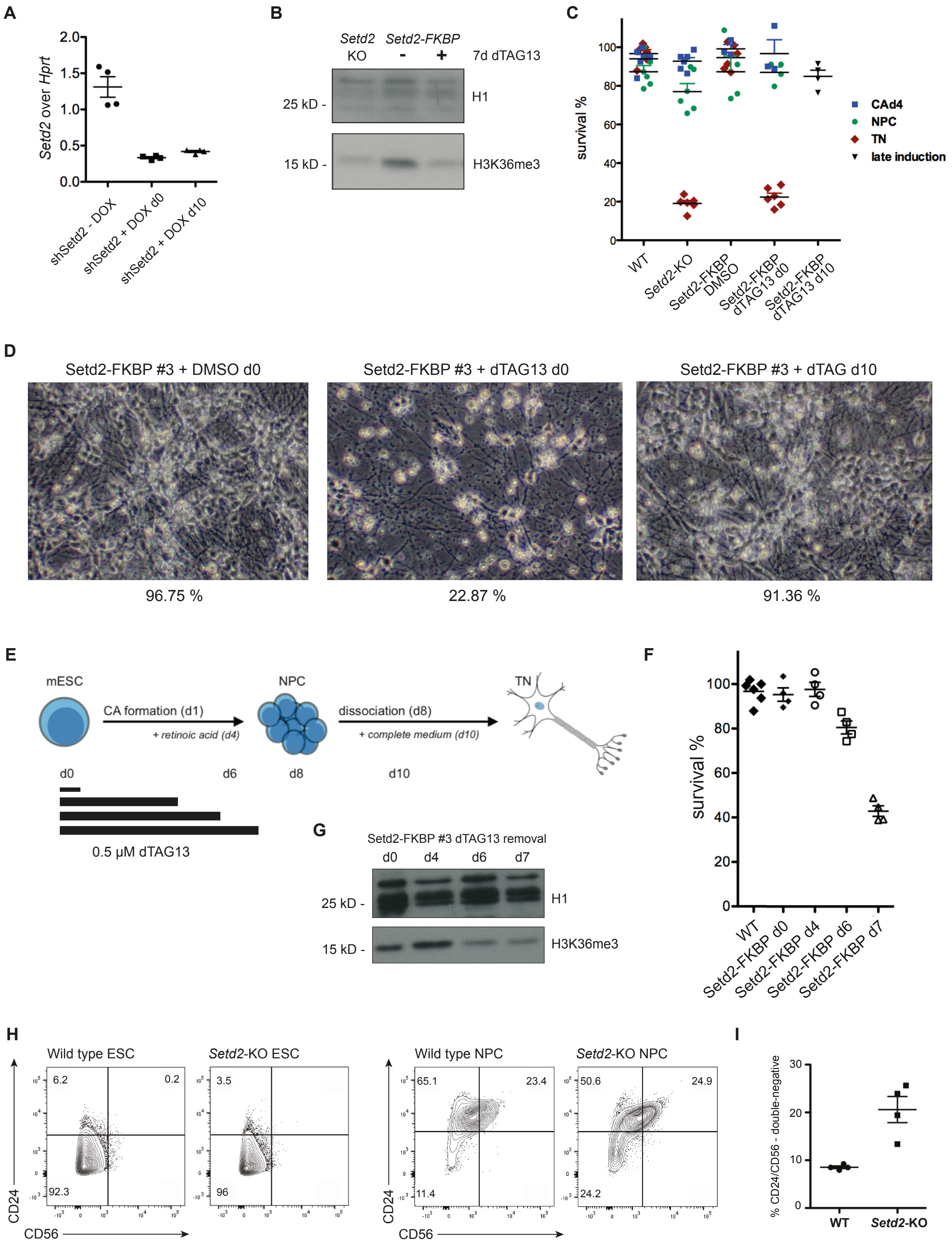

Appendix Figure S5. (A) RT qPCR of *Setd2* in Tet-inducible *Setd2* knockdown cells, differentiated to terminal neurons (day 14), shows efficient knock-down. Cells were treated with 1 µg/ml doxycycline, always or from day 10 on. *Hprt* served as internal control. **(B)** Western blot analysis of H3K36me3 levels after SETD2-FKBP degradation with 0.5 µM dTAG

for 7 days in ESC. **(C)** Cell count assay using live-dead stain at cellular aggregate stage day 4 (CAd4), neural progenitor cells (NPCs), and terminal neurons (TNs) stage day 14. Depicted are percentages for survival in WT, *Setd2*-KO, and wild type cells harboring endogenously tagged Setd2 with FKBP either constitutively treated with DMSO, 0.5 µM dTAG13 throughout the entire differentiation process, or 0.5 µM dTAG13 after day 10. **(D)** Microscopy images of *Setd2*-FKBP derived neurons at day d14 and at 100x magnification treated either with DMSO, 0.5 µM dTAG13 throughout the entire differentiation process, or 0.5 µM dTAG13 after day 10. Average percentages of survival after dissociation obtained from three independent experiments are indicated. **(E)** Treatment scheme for dTAG13 washout experiments. *Setd2*-FKBP ES cells were cultivated in the presence of 0.5 µM dTAG13 for several passages before starting neuronal differentiation. dTAG13 was washed out at different timepoints (d0, d4, d6, and d7) and survival of neuronal cells was measured as previously described. **(F)** Survival of *Setd2*-FKBP neuronal cells, shown as percentage compared to wild type survival indicates that dTAG13 treatment duration during early steps of neuroprogenitor cell cultivation correlates with reduced survival at the neuronal stage. **(G)** Western blot indicates recovery of H3K36me3 levels measured at day 8 of NPC formation in *Setd2*-FKBP cells after dTAG13 washout at different time points of differentiation. (H) Exemplary FACS analysis for the neuronal surface proteins CD-24 and CD-56 in WT and *Setd2*-KO cells at ESC and NPC stage. Highlighted are percentages of committed cells in CD-24-positive, CD-24/CD-56 double-positive, and uncommitted CD-24/CD-56 double-negative clusters.

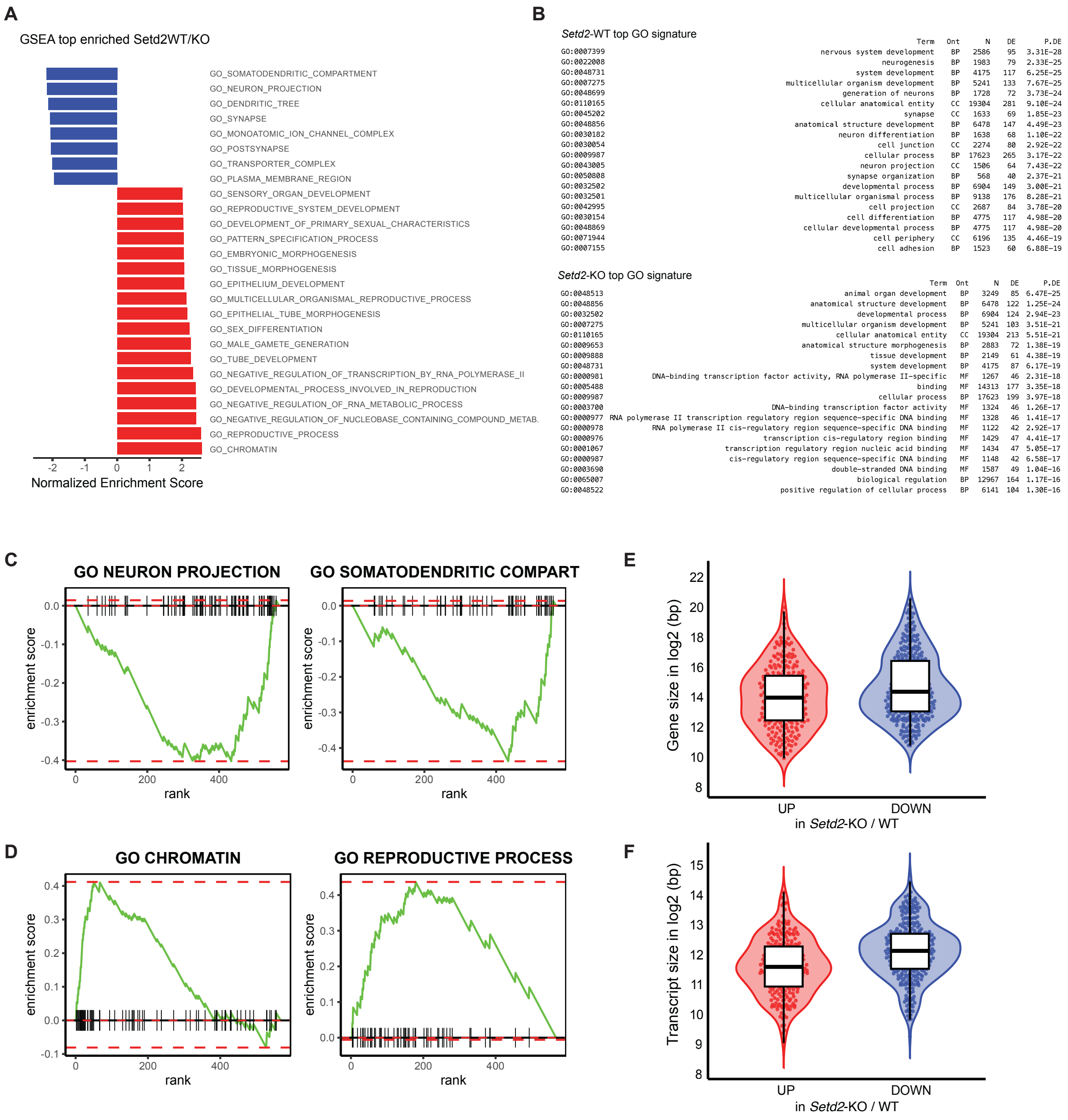

Setd2-WT top GO signature

	Term	Ont	N	DE	P.DE
GO:0007399	nervous system development	BP	2586	95	3.31E-28
GO:0022008	neurogenesis	BP	1983	79	2.33E-25
GO:0048731	system development	BP	4175	117	6.25E-25
GO:0007275	multicellular organism development	BP	5241	133	7.67E-25
GO:0048699	generation of neurons	BP	1728	72	3.73E-24
GO:0110165	cellular anatomical entity	CC	19304	281	9.10E-24
GO:0045202	synapse	CC	1633	69	1.85E-23
GO:0048856	anatomical structure development	BP	6478	147	4.49E-23
GO:0030182	neuron differentiation	BP	1638	68	1.10E-22
GO:0030054	cell junction	CC	2274	80	2.92E-22
GO:0009987	cellular process	BP	17623	265	3.17E-22
GO:0043005	neuron projection	CC	1506	64	7.43E-22
GO:0050808	synapse organization	BP	568	40	2.37E-21
GO:0032502	developmental process	BP	6904	149	3.00E-21
GO:0032501	multicellular organismal process	BP	9138	176	8.28E-21
GO:0042995	cell projection	CC	2687	84	3.78E-20
GO:0030154	cell differentiation	BP	4775	117	4.98E-20
GO:0048869	cellular developmental process	BP	4775	117	4.98E-20
GO:0071944	cell periphery	CC	6196	135	4.46E-19
GO:0007155	cell adhesion	BP	1523	60	6.88E-19

Setd2-KO top GO signature

	Term	Ont	N	DE	P.DE
GO:0048513	animal organ development	BP	3249	85	6.47E-25
GO:0048856	anatomical structure development	BP	6478	122	1.25E-24
GO:0032502	developmental process	BP	6904	124	2.94E-23
GO:0007275	multicellular organism development	BP	5241	103	3.51E-21
GO:0110165	cellular anatomical entity	CC	19304	213	5.51E-21
GO:0009653	anatomical structure morphogenesis	BP	2883	72	1.38E-19
GO:0009888	tissue development	BP	2149	61	4.38E-19
GO:0048731	system development	BP	4175	87	6.17E-19
GO:0000981	DNA-binding transcription factor activity, RNA polymerase II-specific	MF	1267	46	2.31E-18
GO:0005488	binding	MF	14313	177	3.35E-18
GO:0009987	cellular process	BP	17623	199	3.97E-18
GO:0003700	DNA-binding transcription factor activity	MF	1324	46	1.26E-17
GO:0000977	RNA polymerase II transcription regulatory region sequence-specific DNA binding	MF	1328	46	1.41E-17
GO:0000978	RNA polymerase II cis-regulatory region sequence-specific DNA binding	MF	1122	42	2.92E-17
GO:0000976	transcription cis-regulatory region binding	MF	1429	47	4.41E-17
GO:0001067	transcription regulatory region nucleic acid binding	MF	1434	47	5.05E-17
GO:0000987	cis-regulatory region sequence-specific DNA binding	MF	1148	42	6.58E-17
GO:0003690	double-stranded DNA binding	MF	1587	49	1.04E-16
GO:0065007	biological regulation	BP	12967	164	1.17E-16
GO:0048522	positive regulation of cellular process	BP	6141	104	1.30E-16

Appendix Figure S6. (A) Gene set enrichment analysis (GSEA) for differentially expressed genes indicated a reduction of neuronal gene expression in *Setd2*-deficient NPCs. Shown are the top-most enriched terms. **(B)** Gene ontology (GO) term analysis for differentially expressed genes in WT and *Setd2*-deficient NPCs. Shown are the top-most enriched terms. **(C-D)** Top two GO term enrichment scores for (C) downregulated and (D) upregulated genes in *Setd2*-KO cells. **(E-F)** Violin-box plots showing gene size (E) and transcript length (F) differences for up- and downregulated genes in *Setd2*-KO versus wild-type NPCs.

	UP	DOWN	Total
Skipped exon (SE)	33	73	8073
Alternative 5' splice site (A5SS)	4	7	1060
Alternative 3' splice site (A3SS)	8	9	2003
Mutually exclusive exons (MXE)	4	8	4873
Retained intron (RI)	8	78	2099
ALL	57	175	18108

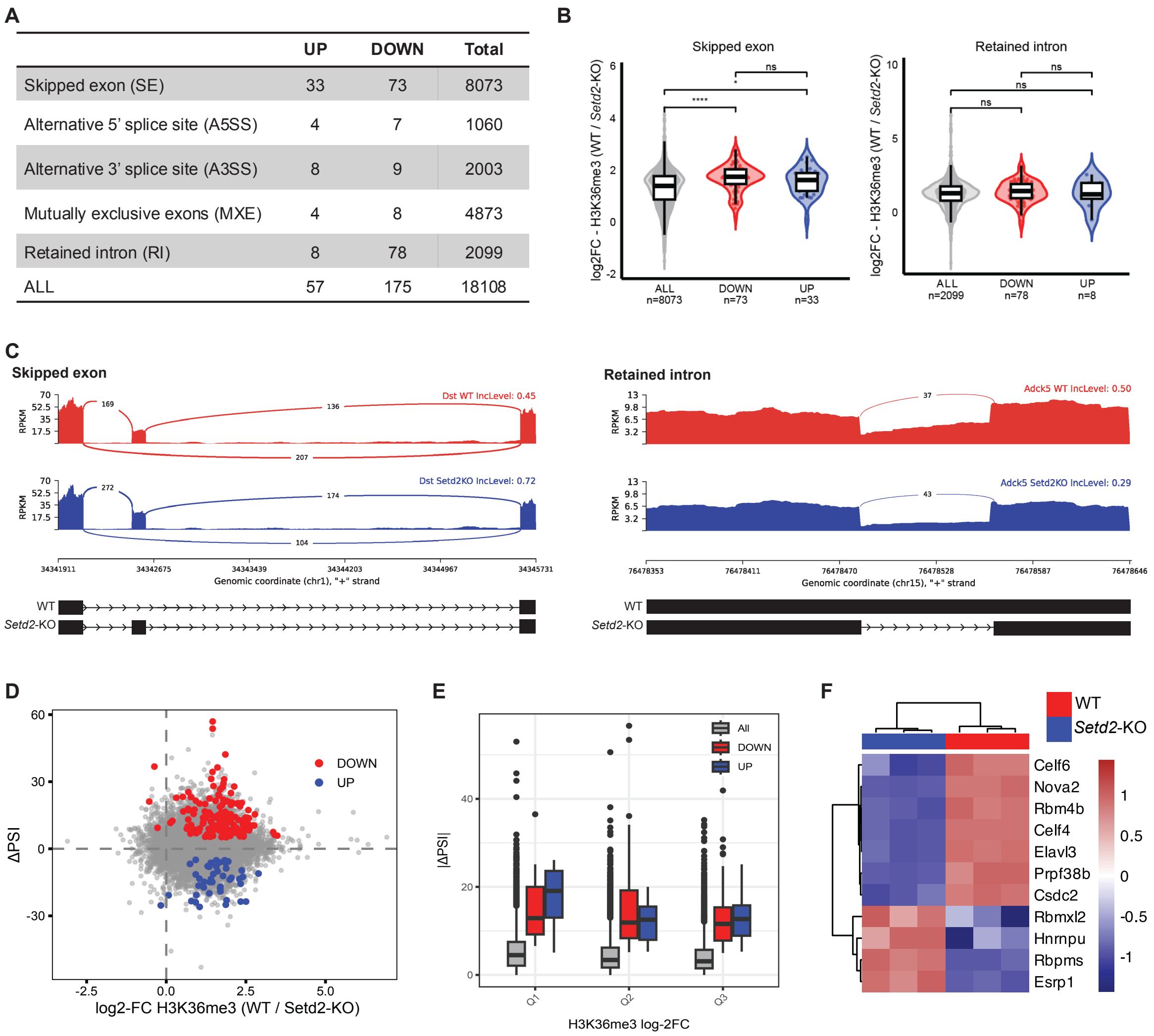

Appendix Figure S7. (A) Summary table of alternative splicing events identified by rMATS in Setd2-KO versus WT NPCs across five major alternative splicing categories. UP and DOWN refer to significantly increased or decreased levels of a splicing event in Setd2-KO cells relative to WT with ΔPSI (percent spliced in) ≥5% and FDR <0.01. Total corresponds to the overall number of events detected by rMATS across the dataset, irrespective of significance. **(B)** Violin plots showing log2 fold-change in H3K36me3 (WT/Setd2-KO) over skipped exons and retained introns for all detected events (ALL), decreased (DOWN), or increased in Setd2-KO (UP). Boxplots indicate the median and interquartile range. Whiskers extend to 1.5x IQR. Differences between groups were tested using Wilcoxon rank-sum test (*p<0.05;**** p <0.0001; ns, not significant). **(C)** Representative sashimi plots illustrating examples of a skipped exon event and a retained intron event, with junction read counts and inclusion levels (IncLevel) shown for WT (red) and Setd2-KO (blue). **(D)** Scatter plot of ΔPSI (percent spliced in) versus log2 fold-change of H3K36me3 (WT/Setd2-KO) over the spliced region. Significantly altered events are highlighted in red (DOWN) or blue (UP). **(E)** Boxplots of ΔPSI (percent

spliced in) for significant alternative splicing events stratified into quartiles based on the log2 fold-change of H3K36me3 signal (WT/Setd2-KO) measured over the affected regions. **(F)** Heatmap of differentially expressed RNA-binding proteins and splicing regulators in WT and Setd2-KO NPCs, showing altered expression patterns that may contribute to splicing changes. Expression values were obtained as TMM-normalized log2 counts per million from RNA-seq and visualized as relative expression values (z-scores). Hierarchical clustering was applied to both genes and samples.

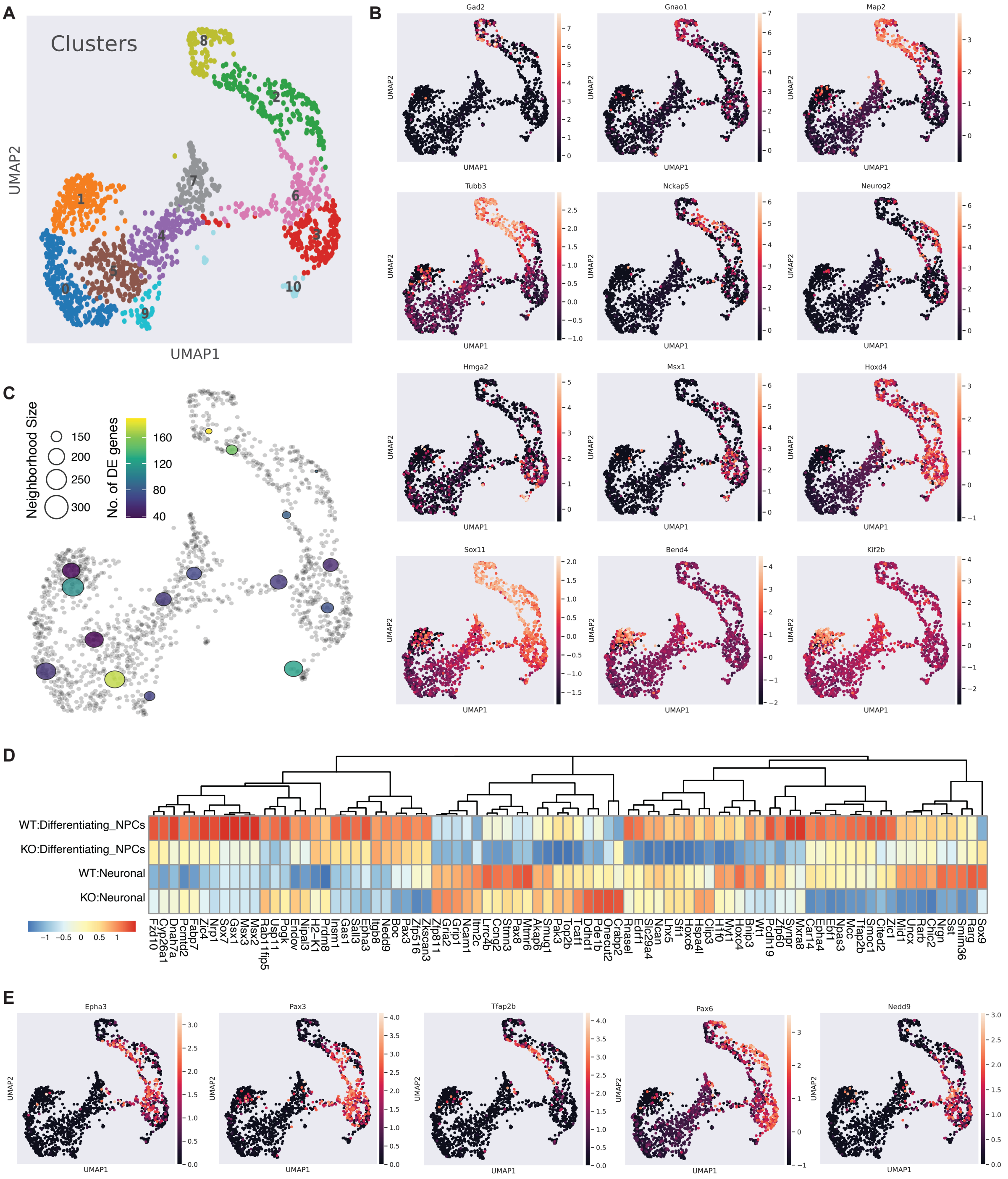

Appendix Figure S8. **(A)** UMAP projection and unsupervised clustering of cells based on single-cell RNA seq. **(B)** Examples of marker genes with specific expression in the individual clusters. **(C)** Unsupervised local neighborhood-based differential gene expression analysis from transcriptionally similar cells identifies 16 separate clusters. Shown are the neighborhood size and the number of differentially expressed genes per cluster over the UMAP projection. **(D)** Heatmap indicating 88 genes with differential gene expression between wild type and *Setd2*-KO cells in the differentiating NPC and neuronal annotations. **(E)**

Examples of differentially expressed genes between wild type and *Setd2*-KO NPC and neuronal cells.

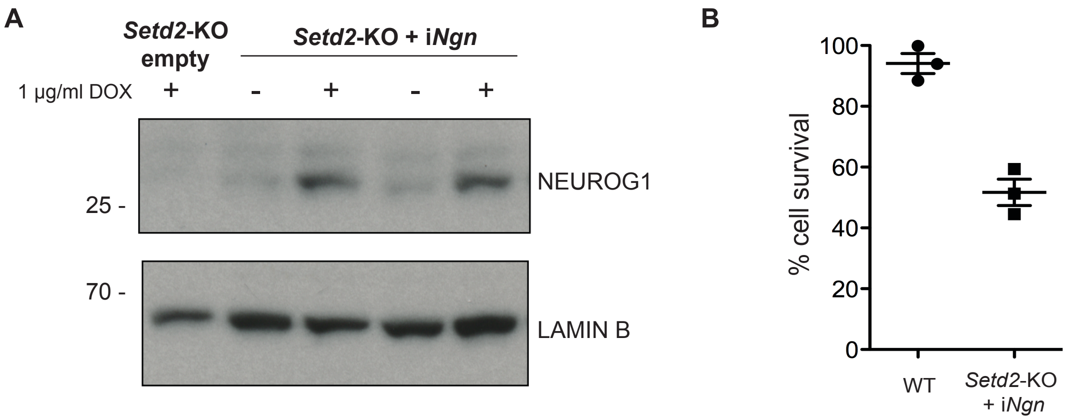

Appendix Figure S9. (A) Immunoblot analysis for Neurogenin 1 (NEUROG1) in two Tet-inducible *neurogenin2-2A-neurogenin1* (i*Ngn*) ESC-derived neurons in comparison to the parental *Setd2*-KO cell line. LAMIN B1 serves as loading control. Cells were treated either without or always with 1 µg/ml doxycycline (DOX) during *in vitro* differentiation. **(B)** Shown are percentages of survival at d14 of three independent differentiation rounds.

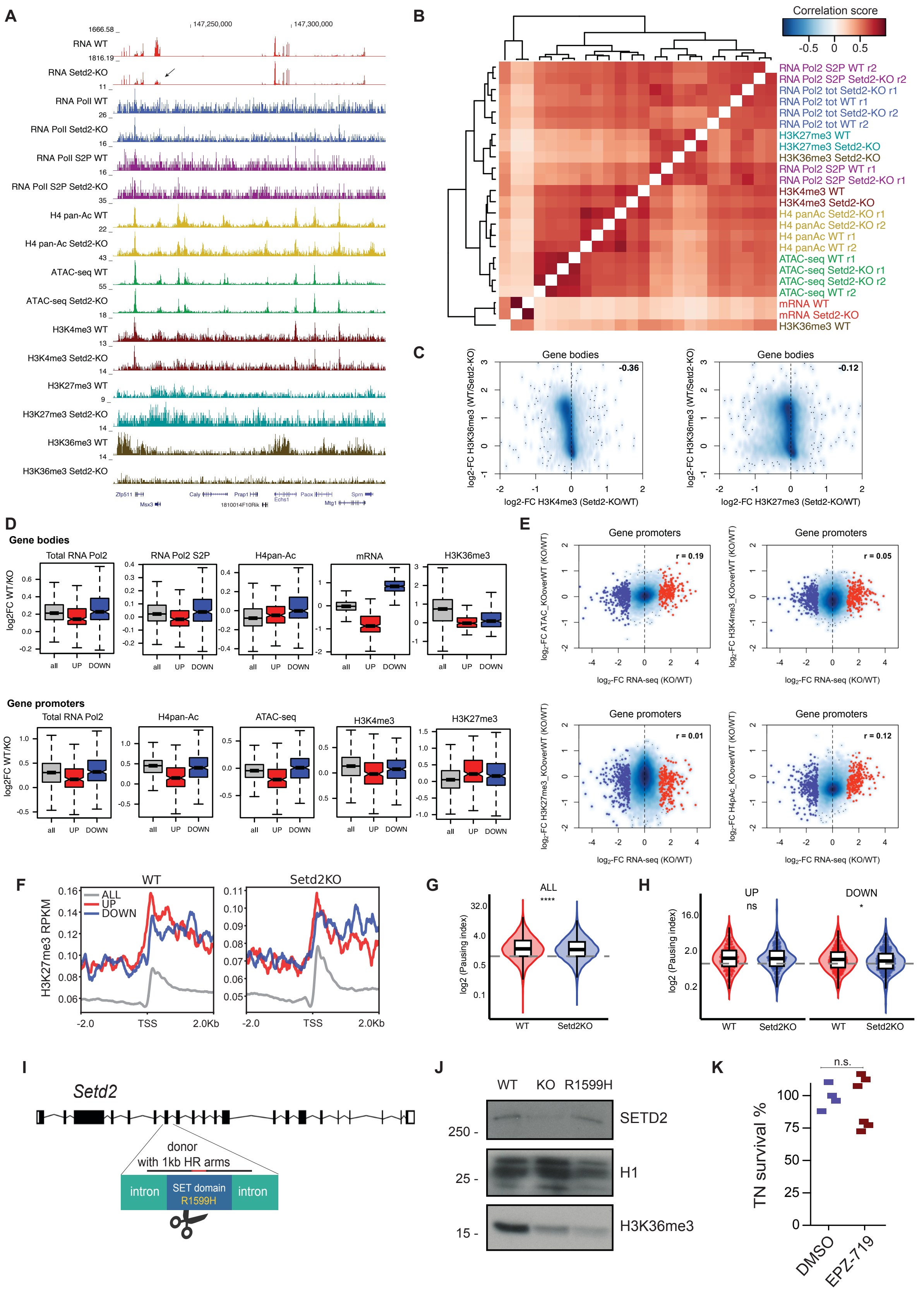

Appendix Figure S10. (A) Representative genome browser view exemplifying ChIP-seq signals of various chromatin marks and ATAC-seq signal between wild-type and *Setd2*-KO NPCs. Shown are read counts per 100 bp for ChIP-seq samples. Arrow indicates downregulation of

the neuronal homeodomain transcription factor *Msx3*. **(B)** Clustered heatmap presenting correlation scores between various ChIP- and ATAC-seq levels wild-type and *Setd2*-KO NPCs (in replicates). Datapoints are 1kb-sized genomic intervals covering the entire genome. **(C)** Scatterplots comparing the measured H3K4me3 and H3K27me3 changes in *Setd2*-KO over WT NPCs with H3K36me3 levels at gene bodies measured in WT cells. Pearson's correlation coefficient is shown. **(D)** Boxplots indicating the differences in various chromatin marks as function of gene expression differences between wild-type and *Setd2*-KO NPCs at gene bodies (top) and promoters (bottom). **(E)** Scatterplot showing changes in gene expression measured in *Setd2*-KO over WT NPCs and their relation to histone modification and chromatin accessibility changes at their gene promoters in *Setd2*-KO over WT NPCs. Pearson's correlation coefficient is shown. Red and blue dots denote significantly up and down-regulated genes in *Setd2*-KO NPC, respectively. **(F)** Metaprofile plots of H3K27me3 ChIP-seq data obtained from wild type and *Setd2*-KO NPCs at genes up- and down-regulated in absence of SETD2 in red and blue, respectively. Grey line shows H3K27me3 levels at all genes, independent of their transcriptional change. **(G-H)** Violin-box plots showing pausing index calculated as promoter / genebody signal ratio based on RNA Pol II ChIP-seq data from NPCs for (G) all genes and (H) for up- and downregulated genes in *Setd2*-KO versus wild-type NPCs. Boxplots indicate the median and interquartile range. Whiskers extend to 1.5x IQR. Differences between groups were tested using Wilcoxon rank-sum test (*$p<0.05$;**** $p <0.0001$; ns, not significant). **(I)** Schematic overview for the generation of an mESC line harboring an endogenous mutation at the catalytic SET domain of *Setd2* leading to an amino acid change from arginine to histidine at position 1599 (R1599H). **(J)** Western blot indicating a reduction in H3K36me3 in presence of the *Setd2* R1599H mutation. Histone H1 serves as loading control. Note that the antibody used to detect H3K36me3 has a residual affinity for H3K36me2. **(K)** Survival of wild type neuronal cells after continuous treatment with either DMSO or the SETD2 inhibitor EPZ-719 (1µM) during the entire differentiation process.

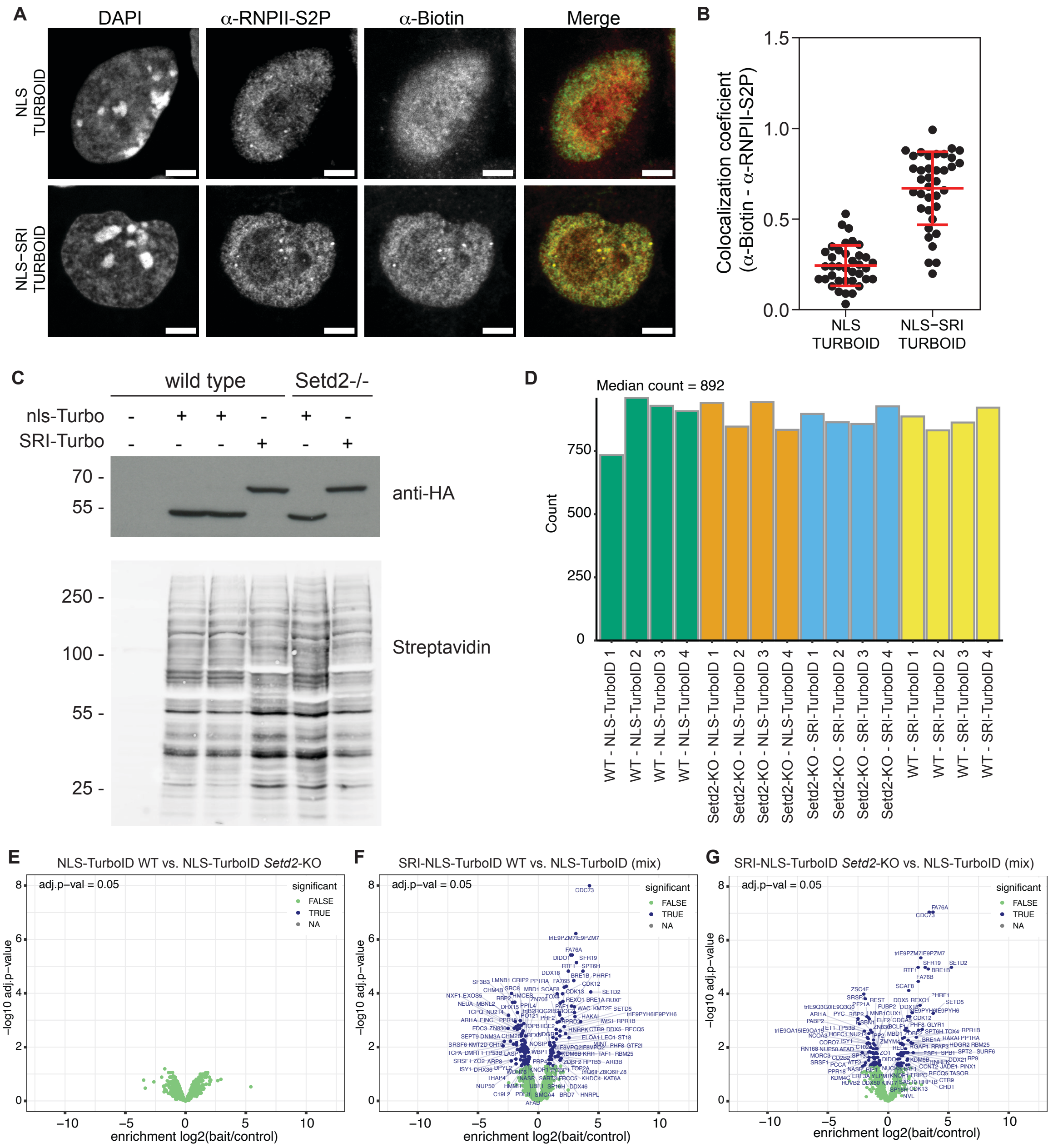

Appendix Figure S11. (A) Representative immunofluorescence images of fixed mESCs pre incubated with Biotin for 1h. Cells were co-stained with anti-Biotin and anti RNAPII-S2P. Nuclei were visualized with DAPI. Merged cells stained with anti-Biotin (red) and anti RNAPII-S2P (green) are shown. Scale bar = 5 µm. **(B)** Mander's correlation coefficient showing the colocalization of Biotin with RNAP2-S2P. Bars represent mean ±SD of 37 nuclei. Experiment was repeated two times. **(C)** Western blot analysis shows HA-tagged SRI-TurboID and NLS-TurboID protein expression in wild type and *Setd2*-KO cells (top). Increased protein biotinylation upon presence of TurboID proteins is indicated by Streptavidin detection of biotinylated proteins (bottom). **(D)** Number of individual proteins detected in four replicates

after proximity biotinylation in the respective cell lines and detection via mass spectrometry. **(E)** Volcano plot showing results comparing the nuclear TurboID controls between WT and *Setd2*-KO NPCs. **(F-G)** ChromID results showing enriched proteins using SRI-TurboID over the pooled NLS-TurboID samples in wild type (F) and *Setd2*-KO NPC cells (G). Statistically enriched proteins are indicated (FDR-corrected two-tailed *t*-test: FDR = 0.01, s0 = 0.1, $\log_2$ FC > 0, *n* = 4 independent replicates).

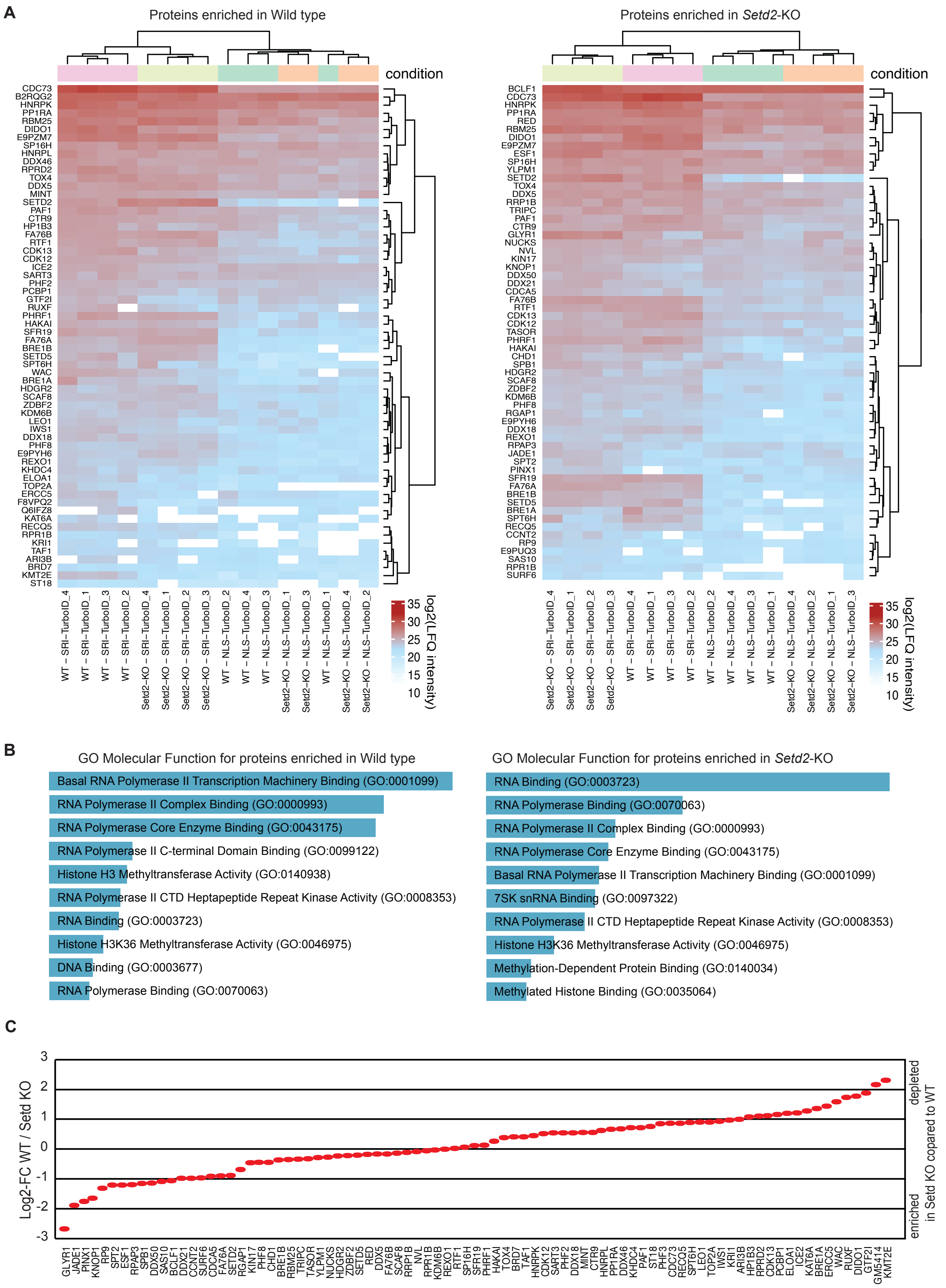

Appendix Figure S12. (A) Heat-map representation of proteins significantly enriched in wild type or *Setd2*-KO NPCs over the pooled NLS-TurboID sample. Shown are the log2-transformed

LFQ intensities obtained from all samples and replicates. **(B)** Bar plots representing the top ten Molecular Function GO terms summarizing the proteins enriched by the SRI-TurboID in wild type and *Setd2*-KO NPCs. Data was generated using the Enrichr database. **(C)** Direct fold change comparison between WT and *Setd2*-KO cells indicates proteins that show enriched or reduced interactions with elongating RNA Pol II in absence of SETD2. Log2-FC was calculated by subtracting the log2FC enrichments in *Setd2*-KO over pooled NLS-TurboID samples from log2FC enrichments in *WT* over pooled NLS-TurboID. Only proteins that were significantly enriched in wild type and/or *Setd2*-KO cells are shown.

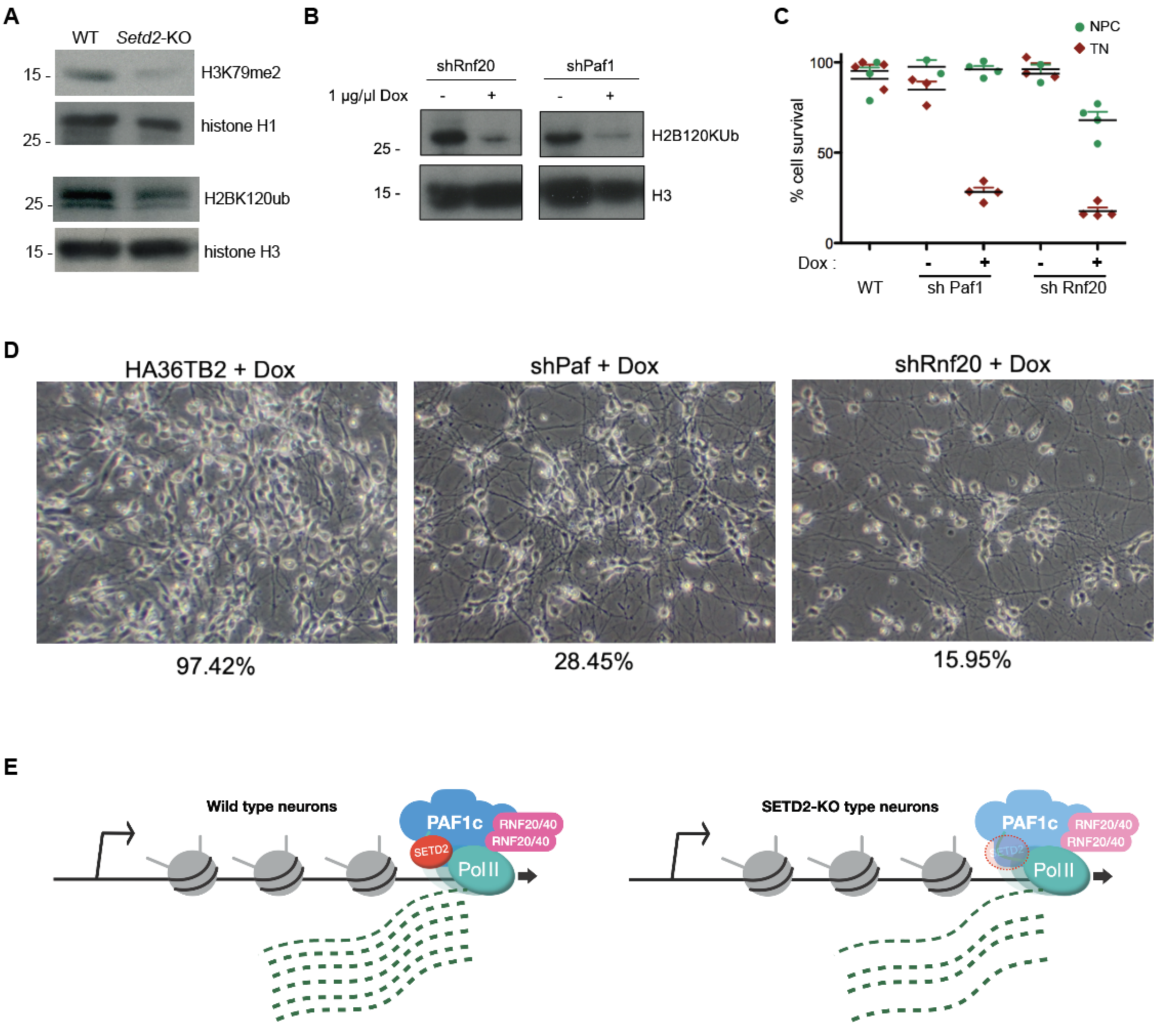

Appendix Figure S13. (A) Immunoblot analysis indicates reduced H3K79me2 and H2BK120Ub levels in *Setd2*-KO NPCs. Histones H1 and H3 served as loading controls. **(B)** Western blot showing reduced H2BK120ub upon Tet-induced expression of sh-RNAs against Rnf20 or Paf1. Histone H3 serves as loading control. **(C)** Cell survival assay using live-dead stain indicated reduced TN survival upon sh-mediated knock-down of Paf1 or Rnf20. Depicted are percentages for survival obtained from four independent replicates at NPC and TN stage. Bars and whiskers indicate means and SD. **(D)** Microscopy images of *in vitro*-derived neurons at day d14 showing the effect of Tet-inducible shRNA against *Paf1* or *Rnf20* on neuronal differentiation. Shown are average percentages of survival after dissociation (plated/attached) of three independent experiments; 100x magnification. Cells were treated with 1 µg/ml doxycycline (DOX) from the start of the differentiation. **(E)** Schematic model summarizing SETD2's catalytically independent role in shaping transcription from neural genes through contributing to interactions between the PAF complex and elongating RNA P2.
