# Supplementary figures and images for: The H3K36me3 methyltransferase SETD2 contributes to PAF1C interactions with RNA Pol II and is required for neuronal differentiation
Source: EMBO J. 2026 Apr 10;45(10):3430–43. doi: 10.1038/s44318-026-00768-2 (PMC13187324; doi:10.1038/s44318-026-00768-2)

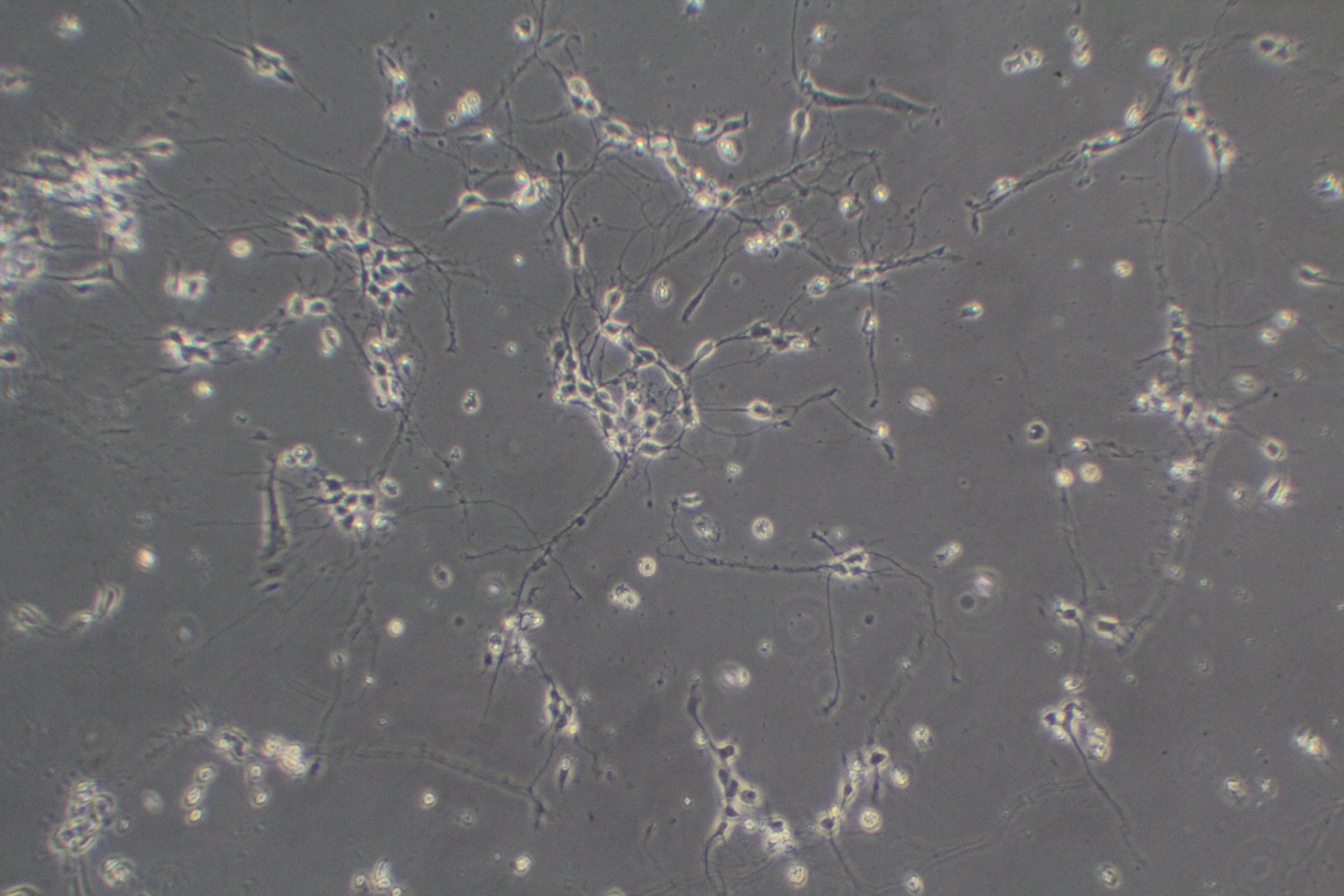

Supplement: Supplementary file 7 — Source data Fig. 1 [file 44318_2026_768_MOESM7_ESM.zip › Figure 1/Fig1c_Setd2_sh1_day0dox.JPG]

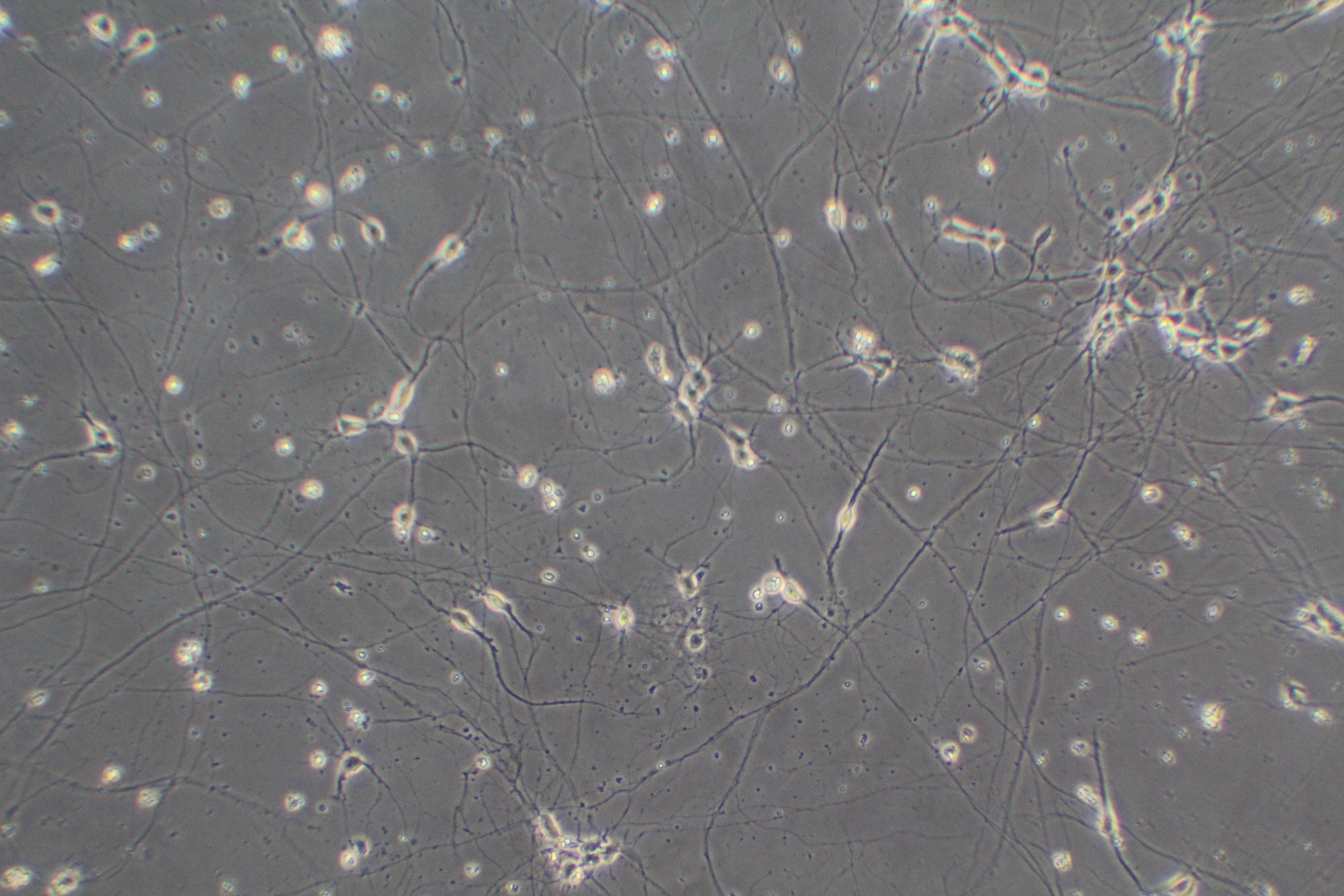

Supplement: Supplementary file 7 — Source data Fig. 1 [file 44318_2026_768_MOESM7_ESM.zip › Figure 1/Fig1b_TN_Setd2KO.JPG]

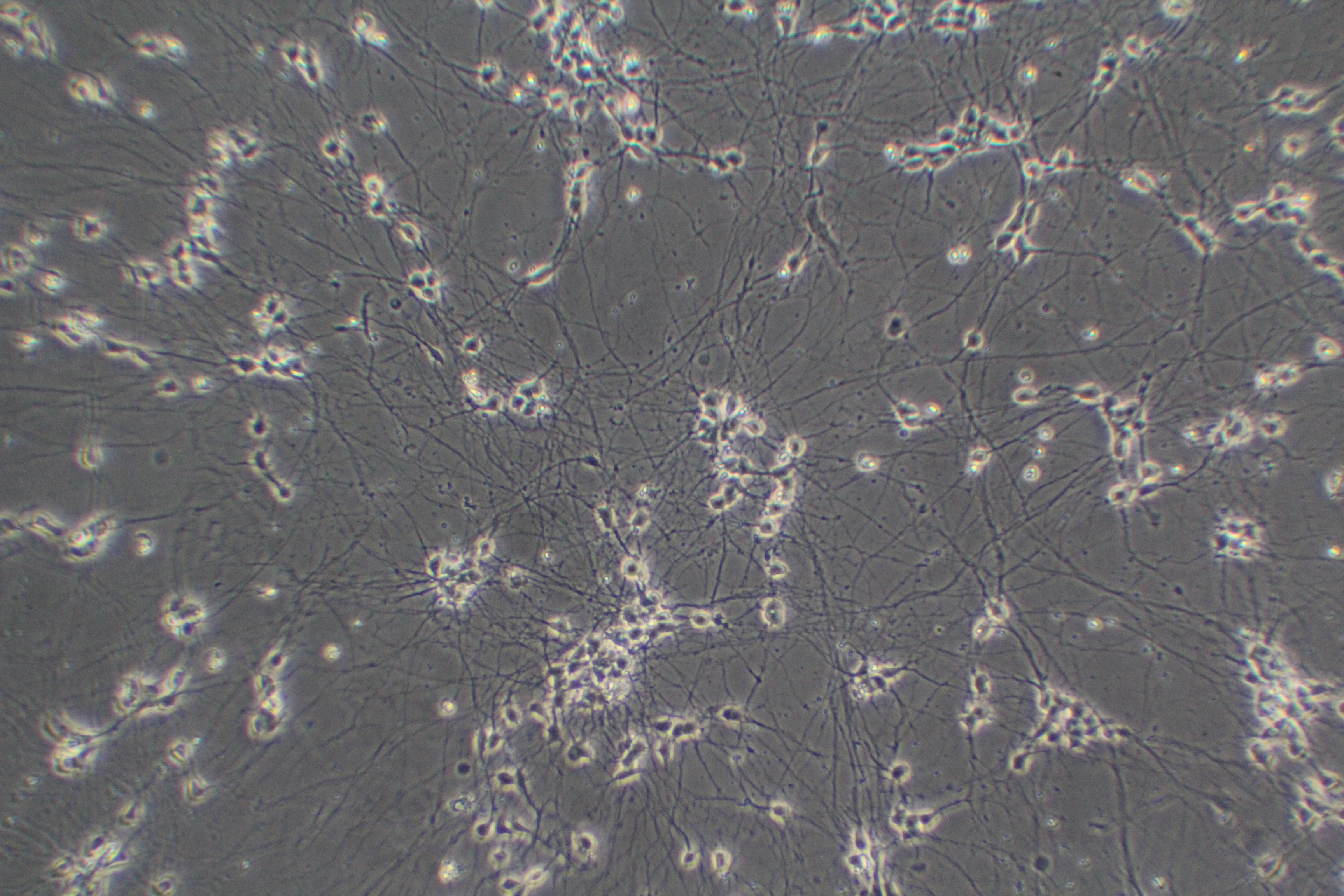

Supplement: Supplementary file 7 — Source data Fig. 1 [file 44318_2026_768_MOESM7_ESM.zip › Figure 1/Fig1c_Setd2_sh1_day10dox.JPG]

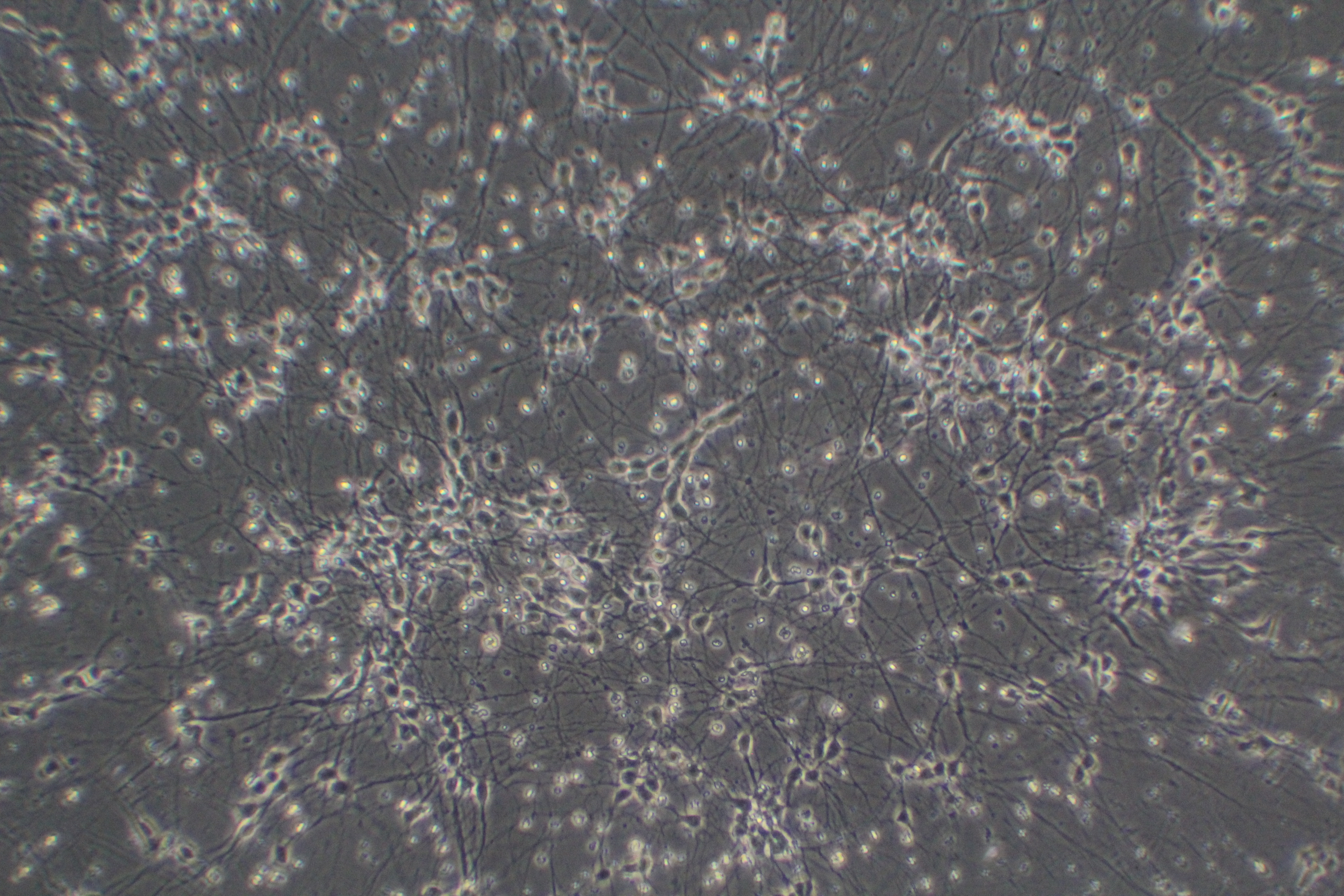

Supplement: Supplementary file 7 — Source data Fig. 1 [file 44318_2026_768_MOESM7_ESM.zip › Figure 1/Fig1b_TN_WT.JPG]

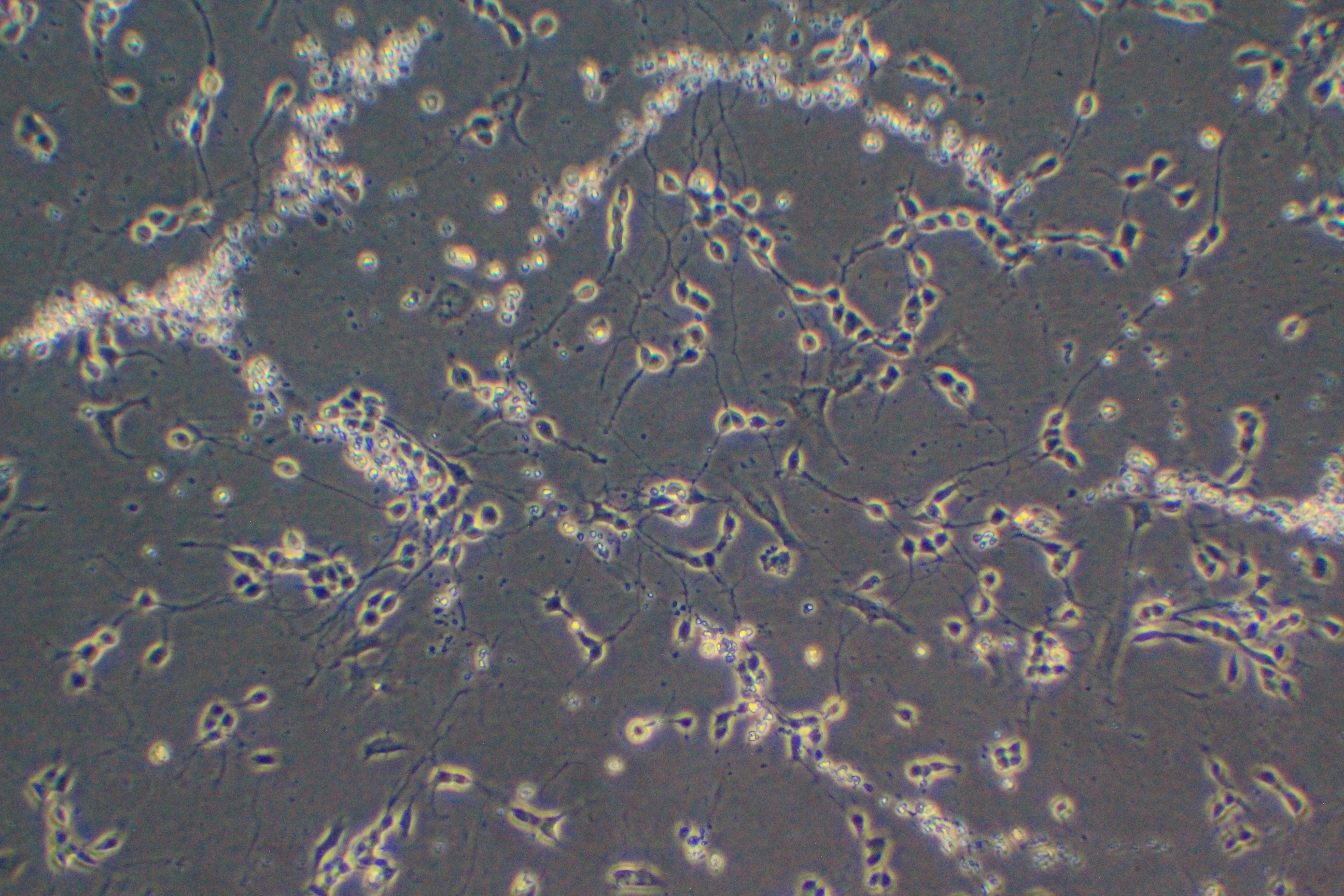

Supplement: Supplementary file 8 — Source data Fig. 3 [file 44318_2026_768_MOESM8_ESM.zip › Figure 3/Fig3b_iNgn-Setd2-KO-withDOX.JPG]

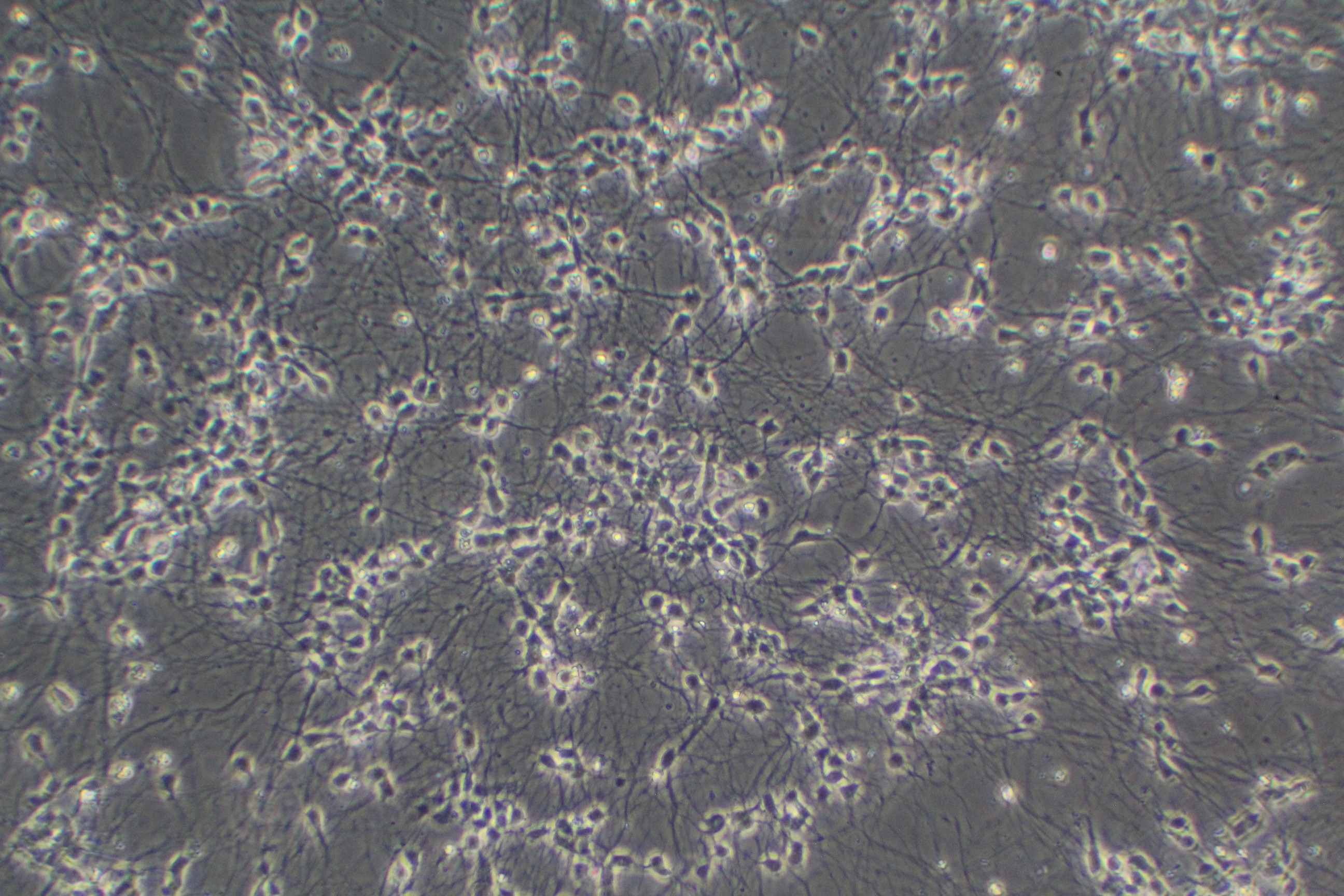

Supplement: Supplementary file 8 — Source data Fig. 3 [file 44318_2026_768_MOESM8_ESM.zip › Figure 3/Fig3b_WT-noDOX.JPG]

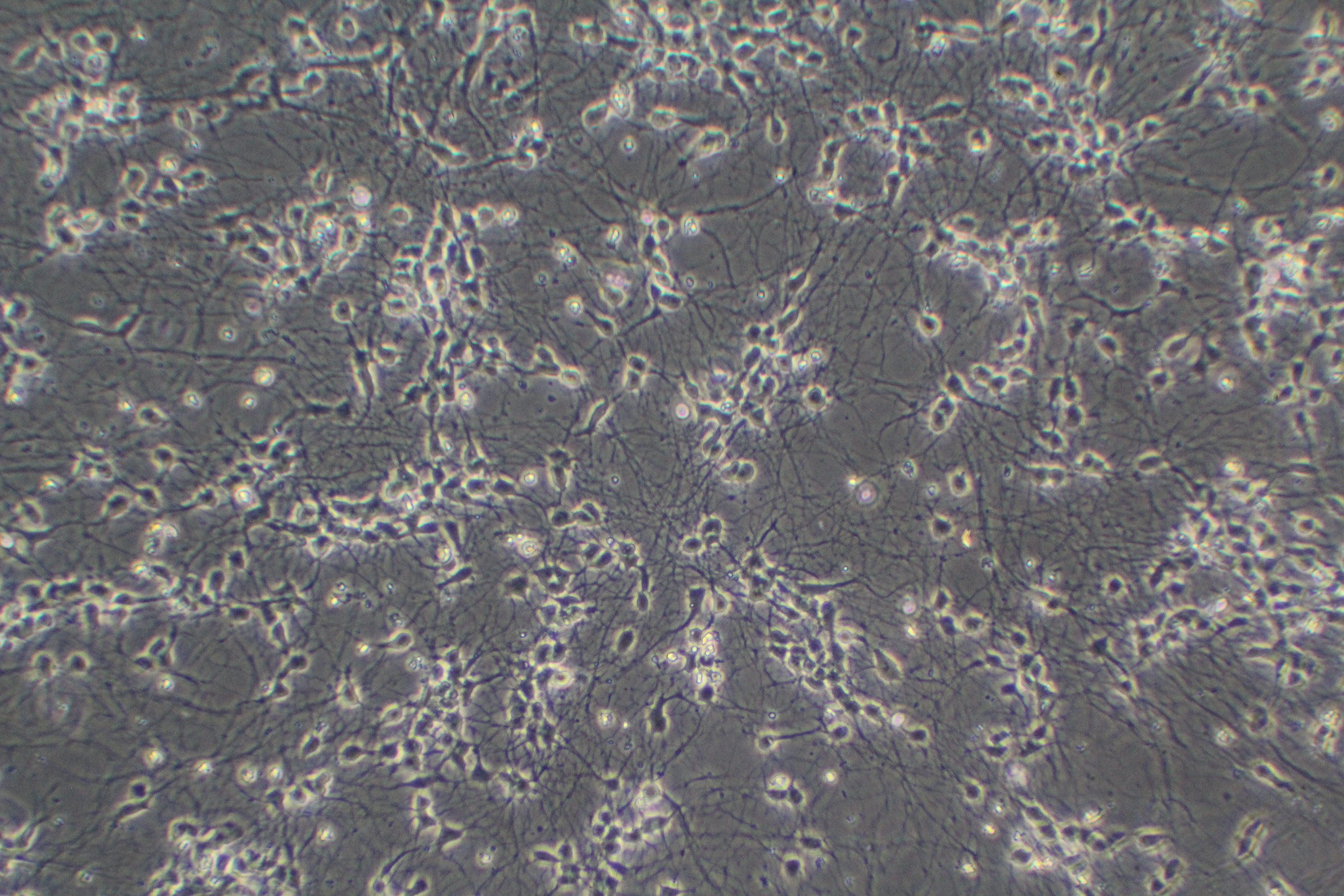

Supplement: Supplementary file 8 — Source data Fig. 3 [file 44318_2026_768_MOESM8_ESM.zip › Figure 3/Fig3b_WT-withDOX.JPG]

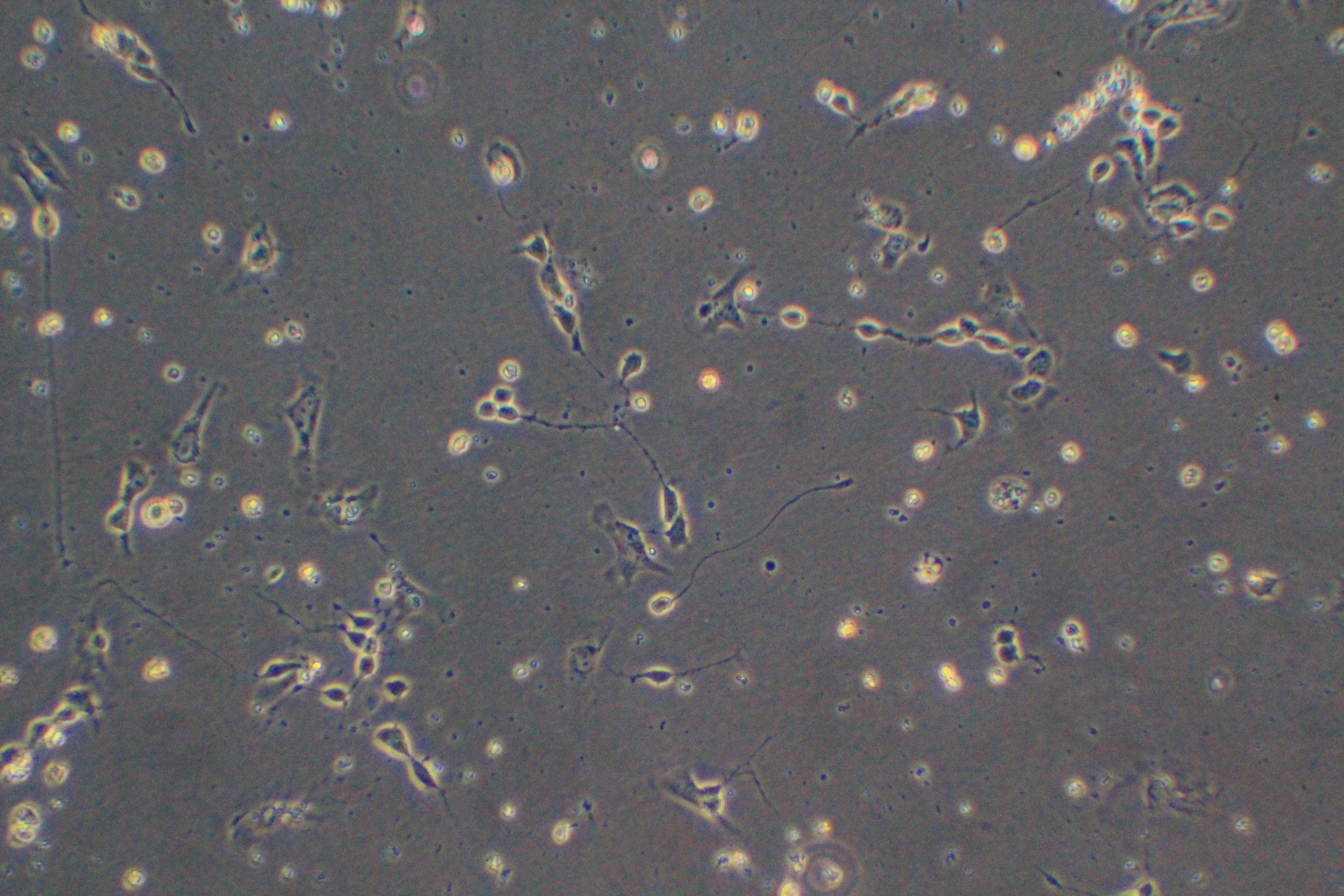

Supplement: Supplementary file 8 — Source data Fig. 3 [file 44318_2026_768_MOESM8_ESM.zip › Figure 3/Fig3b_iNgn-Setd2-KO-noDOX.JPG]
